# VIEWER: an extensible visual analytics framework for enhancing mental healthcare

**DOI:** 10.1093/jamia/ocaf010

**Published:** 2025-01-23

**Authors:** Tao Wang, David Codling, Yamiko Joseph Msosa, Matthew Broadbent, Daisy Kornblum, Catherine Polling, Thomas Searle, Claire Delaney-Pope, Barbara Arroyo, Stuart MacLellan, Zoe Keddie, Mary Docherty, Angus Roberts, Robert Stewart, Philip McGuire, Richard Dobson, Robert Harland

**Affiliations:** Department of Biostatistics & Health Informatics, Institute of Psychiatry, Psychology & Neuroscience, King’s College London, London SE5 8AF, United Kingdom; South London and Maudsley NHS Foundation Trust, London SE5 8AZ, United Kingdom; Department of Biostatistics & Health Informatics, Institute of Psychiatry, Psychology & Neuroscience, King’s College London, London SE5 8AF, United Kingdom; South London and Maudsley NHS Foundation Trust, London SE5 8AZ, United Kingdom; South London and Maudsley NHS Foundation Trust, London SE5 8AZ, United Kingdom; South London and Maudsley NHS Foundation Trust, London SE5 8AZ, United Kingdom; Department of Psychological Medicine, Institute of Psychiatry, Psychology & Neuroscience, King’s College London, London SE5 8AF, United Kingdom; Department of Biostatistics & Health Informatics, Institute of Psychiatry, Psychology & Neuroscience, King’s College London, London SE5 8AF, United Kingdom; South London and Maudsley NHS Foundation Trust, London SE5 8AZ, United Kingdom; South London and Maudsley NHS Foundation Trust, London SE5 8AZ, United Kingdom; South London and Maudsley NHS Foundation Trust, London SE5 8AZ, United Kingdom; South London and Maudsley NHS Foundation Trust, London SE5 8AZ, United Kingdom; South London and Maudsley NHS Foundation Trust, London SE5 8AZ, United Kingdom; Department of Biostatistics & Health Informatics, Institute of Psychiatry, Psychology & Neuroscience, King’s College London, London SE5 8AF, United Kingdom; South London and Maudsley NHS Foundation Trust, London SE5 8AZ, United Kingdom; Department of Psychological Medicine, Institute of Psychiatry, Psychology & Neuroscience, King’s College London, London SE5 8AF, United Kingdom; Department of Psychiatry, University of Oxford, Oxford OX3 7JX, United Kingdom; Department of Biostatistics & Health Informatics, Institute of Psychiatry, Psychology & Neuroscience, King’s College London, London SE5 8AF, United Kingdom; Institute of Health Informatics, University College London, London NW1 2DA, United Kingdom; Health Data Research United Kingdom, London NW1 2BE, United Kingdom; South London and Maudsley NHS Foundation Trust, London SE5 8AZ, United Kingdom

**Keywords:** electronic health record, visual analytics, natural language processing, health informatics, mental health

## Abstract

**Objective:**

A proof-of-concept study aimed at designing and implementing Visual & Interactive Engagement With Electronic Records (VIEWER), a versatile toolkit for visual analytics of clinical data, and systematically evaluating its effectiveness across various clinical applications while gathering feedback for iterative improvements.

**Materials and Methods:**

VIEWER is an open-source and extensible toolkit that employs natural language processing and interactive visualization techniques to facilitate the rapid design, development, and deployment of clinical information retrieval, analysis, and visualization at the point of care. Through an iterative and collaborative participatory design approach, VIEWER was designed and implemented in one of the United Kingdom’s largest National Health Services mental health Trusts, where its clinical utility and effectiveness were assessed using both quantitative and qualitative methods.

**Results:**

VIEWER provides interactive, problem-focused, and comprehensive views of longitudinal patient data (*n* = 409 870) from a combination of structured clinical data and unstructured clinical notes. Despite a relatively short adoption period and users’ initial unfamiliarity, VIEWER significantly improved performance and task completion speed compared to the standard clinical information system. More than 1000 users and partners in the hospital tested and used VIEWER, reporting high satisfaction and expressed strong interest in incorporating VIEWER into their daily practice.

**Discussion:**

VIEWER provides a cost-effective enhancement to the functionalities of standard clinical information systems, with evaluation offering valuable feedback for future improvements.

**Conclusion:**

VIEWER was developed to improve data accessibility and representation across various aspects of healthcare delivery, including population health management and patient monitoring. The deployment of VIEWER highlights the benefits of collaborative refinement in optimizing health informatics solutions for enhanced patient care.

## Introduction

As the volume of information in patient records continues to grow, clinicians face an overwhelming amount of complex raw data, which can exceed the capacity of human cognition to process without error.^1^ This issue of information overload has been widely noted across various care settings, including both primary and secondary care, which may result in diagnostic or treatment errors.^2^^,^^3^ Moreover, the growing demand for health information exchange among healthcare providers, and between providers and partners such as public health agencies and regulators, has made patient data review and administrative reporting increasingly complex and time-consuming.^4^ This is particularly evident in mental healthcare, where patients often require ongoing mental health support and coordinated care for physical comorbidities and social support.^5^^,^^6^ As a result, clinicians—including psychiatrists, psychologists, pharmacists, nurses, and professionals offering occupational and social support in mental health services—are forced to spend valuable time assembling disparate data points to create a coherent view for a patient’s care, which can lead to inefficiency and delayed care.^7^

Although electronic health records (EHRs) are designed to help clinicians manage information at the point of care, they often fail to present information in a format that offers effective cognitive support and mitigates information overload.^7^ This is largely because EHR systems mainly focus on recording information for individual patients and present data in tabular views or static text formats, with limited capability to highlight underlying trends in a patient’s disease progression, reveal similarities within a team’s caseload, or facilitate longitudinal health monitoring on a population scale.^8^ The need for better methods to manage and present increasingly complex information in EHRs has been long recognized.^9^^,^^10^ Pioneering work in the 1990s introduced graphical summaries of test results and treatment data to enhance the presentation of patient status and reduce the burden of information overload.^11^ Since then, research in this area has advanced by incorporating diverse datasets, including patient-generated data^8^^,^^12^ and knowledge bases,^13^ alongside emerging technologies, for example, interactive visualizations,^14^ statistical and machine learning analytics,^8^ to enhance the effectiveness and usability of visual analytics in healthcare. These advancements have been applied across various domains, including patient data summarization,^7^^,^^15^ cohort search,^16^ care quality improvement,^17^ patient flow analysis,^18^ population health management,^19^ and disease- or setting-specific pathway management.^18^^,^^20^^,^^21^ Visual analytics has emerged as a powerful tool for converting complex health data into intuitive and visually compelling presentations, enabling the extraction of valuable insights from big data and supporting informed clinical decision-making.^22^

However, existing research has mainly focused on physical healthcare settings, with limited attention given to mental healthcare.^23^ Compared to physical health, visual analysis of mental health data presents greater complexity for 2 key reasons. First, mental health conditions often encompass both medical, psychological, and social dimensions,^24^ requiring a broader range of data to achieve a comprehensive assessment of an individual’s mental health. This highlights the need of an extensible toolkit that can facilitate the rapid design, development, and implementation of visual analytics across diverse data sources and types in mental healthcare. Second, treating mental health conditions typically involves a combination of interventions, including medication, psychotherapy, lifestyle changes, and social support services.^6^ Unlike physical conditions, where structured data (such as blood assays and other characterizations) is more salient, mental health assessments also rely on vast patient-reported information as quantitative measures to evaluate various aspects of mental health, including mental health presentations, relevant contextual factors, interventions, and outcomes.^25^ Such information is often documented in unstructured text, such as clinical notes and correspondences.^26^ Integrating this unstructured information into visual analytics pipelines is more challenging compared to structured data such as numerical or categorical variables. Thus, tools developed for physical health management in previous studies may not effectively process and represent mental healthcare data.

Another notable limitation of many previous studies is their generalizability. First, most studies have focused on a specific clinical task^7^^,^^16^ or disease.^18^^,^^27^ However, in numerous clinical settings, patients rarely present with a single disease or single risk factor that may affect their health.^28^ There is a lack of integrated visual-analytics solutions that can systematically address the varied challenges faced by different healthcare partners, including clinicians and patients.^22^ Second, existing solutions are often developed using proprietary or custom tools internally developed by hospitals or EHR system providers,^7^^,^^27^ leading to limited interoperability with other tools and restricted applicability in different settings.^8^ Finally, for visual-analytics solutions to achieve better outcomes and effective adoption, they should be user-friendly, require minimal training, offer evidence-based recommendations, and integrate smoothly into clinicians’ workflows.^28^ Despite extensive focus on technical development in previous studies, there has been little effort to systematically develop and evaluate these solutions within the complex workflows of clinicians, nurses, and other health workers while considering their varied clinical priorities and patient needs.^29^

In this work, we present VIEWER (Visual & Interactive Engagement With Electronic Records), an open-source, cost-effective, and extensible toolkit created for the rapid design, development, and deployment of clinical information retrieval, analysis, and visualization for supporting clinical decision making. VIEWER is an EHR-agnostic framework that utilizes distributed information extraction pipelines, leveraging natural language processing (NLP) methods and open-source visualization techniques to enable comprehensive search and visual analytics of a comprehensive health record from both structured and unstructured patient data within a health institution, rather than a curated dataset for a specific disease or patient cohort. We also systematically describe our interdisciplinary and collaborative approach to participatory design, where we designed, implemented, and evaluated VIEWER on top of a bespoke EHR system within one of the largest National Health Services (NHS) Trusts for mental health in the United Kingdom, aimed at addressing the diverse challenges faced by multiple healthcare partners. The partners included: (i) clinicians who need to synthesize disparate data to understand a patient’s condition within the context of their medical history, (ii) managers who require data-driven insights to optimize resource allocation and identify unmet needs, (iii) researchers who seek to understand disparities in outcomes across populations, and (iv) patients who wish to utilize their own medical data for self-monitoring. Our evaluation demonstrates the effectiveness of VIEWER in enhancing patient care in real-world clinical use cases and provides valuable insights into working collaboratively with clinical workers, researchers, patients and carers, and informaticians to iteratively refine and optimize informatics solutions for improved patient care.

### Objective

This paper describes the development and implementation of VIEWER, an extensible visual-analytics system for improving clinical data accessibility and presentation for clinical decision support and the system’s proof-of-concept applications within mental health services. We first describe a participatory, iterative, and interdisciplinary collaborative design approach used to develop an initial prototype of VIEWER, implement it to extend the EHR ecosystem at the South London and Maudsley NHS Foundation Trust (SLaM), and continuously refine the ecosystem based on output from user feedback cycles. We then detail the technical components of VIEWER, which include distributed NLP-based information retrieval and extraction modules, and open-source visualization services to provide visually interactive, problem-oriented, and holistic presentations of the patient record. Finally, we conduct evaluations to assess VIEWER’s utility and effectiveness in supporting healthcare professionals across various clinical workflows, such as patient health checks, medication reviews, and caseload management, thereby providing opportunities to identify areas for improvement of the implementation in its future iterations.

## Methods

### Setting

The VIEWER platform was developed within the National Institute for Health and Care Research (NIHR) Maudsley Biomedical Research Centre (BRC) and has been deployed at SLaM, the United Kingdom’s largest mental health NHS Trust. SLaM provides more than 240 secondary mental health services (In the UK, secondary mental health services refer to mental health services in secondary care, typically including hospitals, some psychological wellbeing services, community mental health teams, crisis resolution and home treatment teams, assertive outreach teams, and early intervention teams.) for a local population of 1.3 million residents in South London. In addition, SLaM provides more than 50 national and specialist services for people across the United Kingdom and beyond. Each year, the Trust provides inpatient care for over 5000 people and treat more than 40 000 patients.

As one of the earliest adopter of an EHR system in the United Kingdom, SLaM has used its bespoke EHR system called Electronic Patient Journey System (ePJS) for a centralized management of patient data since 2006. This system stores a comprehensive record of all clinical information recorded throughout a patient’s journey when accessing services from the Trust, including demographic and contact information, details of referrals and transfers, clinical assessments, care plans and medications, clinical activity and reviews, as well as correspondence letters and supporting care notes between healthcare professionals. The record consists of both structured data (eg, numerals, dates, and selection-lists) and unstructured free text (eg, written assessments, progress notes, and correspondence).

Like other EHR systems,^7^ ePJS is a Web-based application designed for entering and reviewing patient information, with a specific focus on displaying plain textual data rather than visual graphics. Patient summaries within a clinician’s caseload are presented in a tabular format, where each row provides a link to a patient’s profile. This profile contains various tabs corresponding to different components of patient details, including clinical notes, laboratory tests, diagnoses, and medication prescriptions. All data from the clinical record are fine-grained information at the individual patient level, with no aggregation at the team/population levels or aggregation of temporal information for the same patient. A basic search function allows users to search based on document type, test type, recording date, patient name, and their unique identifiers, such as the national NHS number or local Trust identifier (patient IDs). However, the clinical data in free-text documents, constituting the majority of the EHR, are less amenable for search queries and less accessible to clinicians.

The NIHR Maudsley BRC, the United Kingdom’s BRC focused on mental health and hosted by SLaM, has a long-standing tradition of clinical research using EHR data. A key infrastructure supporting this research is the Clinical Records Interactive Search (CRIS), a case register and governance model that allows researchers to use de-identified EHR data for various research purposes, including epidemiology, health data linkage, and clinical NLP.^30^ Additionally, the BRC developed CogStack, an open-source platform for clinical data retrieval and extraction that enables semantic search of free-text data, risk alerting, and data visualization to support clinical decision-making.^31^ VIEWER builds on the aforementioned research infrastructure by incorporating additional capabilities from clinically focused user feedback, thereby facilitating large-scale use in clinical practice.

### Interdisciplinary collaboration

VIEWER was initiated by an interdisciplinary team, which consisted of 2 clinicians, 2 computer scientists, and 2 health informaticians, at SLaM in January 2020, aiming to enhance data-driven clinical decision support and promote population health management within the Trust. To ensure that the perspectives, needs, and preferences of all partners are considered, an iterative participatory design approach,^32^^,^^33^ a widely used approach for clinical decision support systems (CDSS),^32^^,^^34^ was adopted throughout the design, development, and deployment stages. This approach involves a collaborative process, whereby all partners engage with each other to co-create and co-improve the development and deployment, with the aim of developing solutions that are usable, useful, and effective.

Our design process included 3 phases. In phase 1, partner needs were identified and discussed. These needs stem from (1) brainstorming sessions within the development team, and (2) feedback obtained from prototype demo sessions and design workshops involving the various partners, including clinical workers. Table 1 summarizes the resulting needs identified from different categories of partners in this phase. Phase 2 involved the design and development of a series of prototypes, leveraging interdisciplinary skills from the development team. The team held routine weekly meetings to identify and prioritize areas for improvement, brainstorm practical solutions, and plan the next iteration of development. Each prototype underwent testing, which involves: (1) validating the accuracy of information presented in VIEWER by comparing it with the raw data in ePJS, particularly for data processed by NLP pipelines such as medication extraction,^35^ (2) assessing the appropriateness of the information’s visualization, including the suitability of visual graph types and labeling, and (3) the accessibility of the visual interface, focusing on logical design, layout coherence, and integration with other system components. A prototype was eventually migrated into a production environment after reaching consensus by all relevant partners. Key results from this phase are showcased through the user interfaces detailed in User Interfaces section. In phase 3, each new prototype was piloted and evaluated in a real clinical setting. During this phase, demos and training materials, including user guides, screenshots, and recorded video tutorials, were provided to pilot teams. The pilot sessions were centered on specific use cases and involved participants with diverse backgrounds, demographics, and areas of expertise. Participation was voluntary from teams that requested involvement or expressed interest in a given use case. No formal selection process was implemented, ensuring organic representation to promote diversity, inclusivity, and equity in engagement. Feedback was actively collected during demos, training sessions, and feedback channels (eg, emails and review sessions) to guide the next iteration of development. The key outcomes from this phase are outlined in the use cases in Clinical Applications section and the system usability findings in Usability and Acceptability section.

**Table 1.** Needs identified for different partners.

Role	Needs
Service director	Detect specific needs within local populations
	Identify how well these needs are being met
	Monitor trends in data over time
	Identify inequalities in access
Team manager	Ensure workloads are being distributed evenly
	Identify specific needs in the caseload and how well these needs are being met
	Identify trends over time to support improvement work
	Have a “single version of the truth” to support transitions of care
	Identify patients on different stages of their care with the team
Clinical worker	Identify patients who may benefit from their input
	Track patient parameters and outcomes to evaluate effectiveness/tolerability of interventions
	Deliver evidence-based interventions based on clinical needs and effectiveness
Care coordinator	Ensure equitable division of time based on needs
	Deliver proactive care based on identified needs
	Ensure continuity of care
	Track patient parameters and outcomes to evaluate effectiveness/tolerability of interventions
Patient and carer	Cross-check service data against their own understanding
	Track their data over time
	Track key metrics for their care against good practice

It is important to highlight that the design process followed an iterative approach. Requests from partners and feedback from end-users were continuously gathered and prioritized by the development team to refine a current version and identify areas for improvement in subsequent versions. This process facilitated ongoing feedback loops, which ultimately led to the creation of a final design that more closely aligned with the users’ needs and expectations.

### Overall architecture

**Figure 1.**
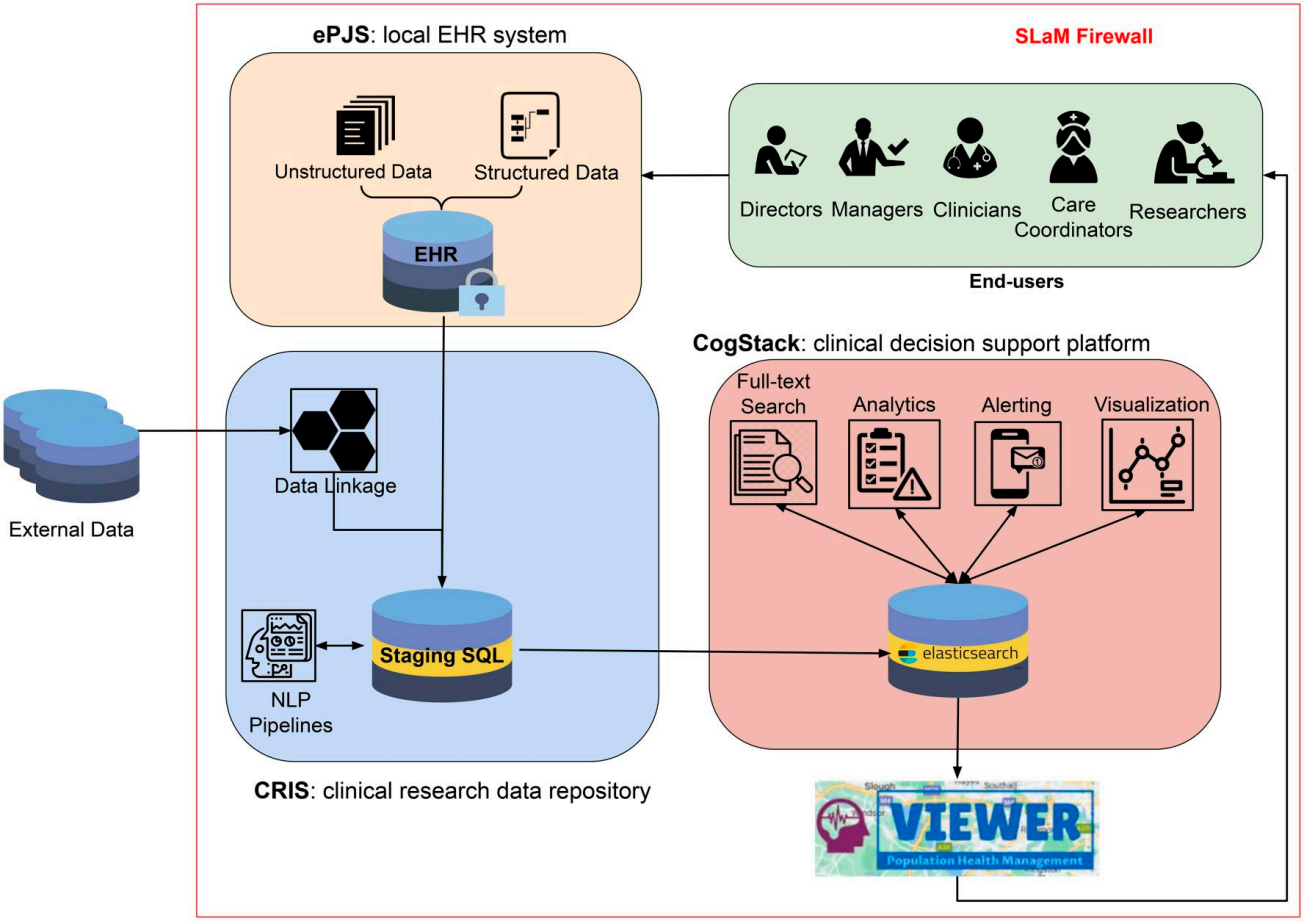
VIEWER system architecture. The diagram presents the architecture of the VIEWER system, a web-based application integrated with three existing systems: (1) ePJS (Electronic Patient Journey System): The EHR system used by SLaM to store patient data; (2) CRIS (Clinical Record Interactive Search): A case register and clinical research data repository that utilizes data linkage and NLP pipelines to develop a data model; and (3) CogStack: An open-source platform for data retrieval and extraction, which aids in clinical decision-making through semantic search, data analytics, visualization, and risk alerting. The architecture also illustrates the flow of patient data. Source EHR data collected at the point of care is processed through data linkage and NLP pipelines within the CRIS system before being ingested into CogStack for visual analytics. The visualized information supports end-users in clinical decision-making, with all decisions, actions, and outcomes subsequently recorded in the source EHR data, completing a feedback loop. All data and pipelines are held securely within the Trust firewall on Azure cloud servers.

Figure 1 illustrates the architecture and pipelines used in VIEWER. Patient data, comprising both structured and unstructured information, are initially collected and recorded in a SQL database using ePJS, SLaM’s EHR system, at the point of care. The updated EHR in ePJS is synchronized daily with a staging SQL database in the CRIS system using Extract, Transform, and Load (ETL) scripts. This staging environment allows the ETL processes to not only handle raw data in ePJS, but also to integrate NLP pipelines for extracting key medical information (eg, symptoms, interventions, outcomes, and contextual factors) from free-text notes,^36^ and data linkage connecting local data with other clinical data sources to enhance the richness of EHR.^37^ The NLP pipelines extract mentions of medical entities, such as symptoms and interventions, from clinical notes using rule-based and machine learning approaches built on the General Architecture for Text Engineering (GATE) software. GATE is a comprehensive suite of tools used for diverse NLP tasks such as text parser, morphology, tagging, and information extraction.^38^ These NLP pipelines have been regularly validated (with precision and recall metrics reported and updated in^36^) and deployed to extract clinical information from routinely collected EHR in CRIS. Details on the development and validation of the NLP applications within CRIS are documented in previous publications^26^^,^^38^^,^^39^ and online resources.^36^ We designed and tested the ETL pipelines based on de-identifiable data in CRIS and plug in these pipelines to raw EHR from ePJS to enable clinical use of data.

The harmonized data model is then ingested into CogStack using customized Python scripts with Elasticsearch Application Programming Interface and Apache NiFi (https://github.com/CogStack/CogStack-NiFi). Interactive visualizations of the ingested data and dashboards are created using the Kibana component in CogStack. We used the open-source versions of Elasticsearch and Kibana from Open Distro (https://opendistro.github.io/for-elasticsearch-docs/) in our implementation. To enhance user experience, a lightweight, customized wrapper application is created, serving as interfaces that logically organize individual dashboards based on clinical use cases. End-users can seamlessly navigate from VIEWER to ePJS to take action (eg, adjusting care plan for a patient) based on insights provided by VIEWER. User authentication and access control are handled by the EHR system using the Lightweight Directory Access Protocol.^40^ For more technical details of each component in the architecture, see our previous papers.^30^^,^^31^

### Data model

**Figure 2.**
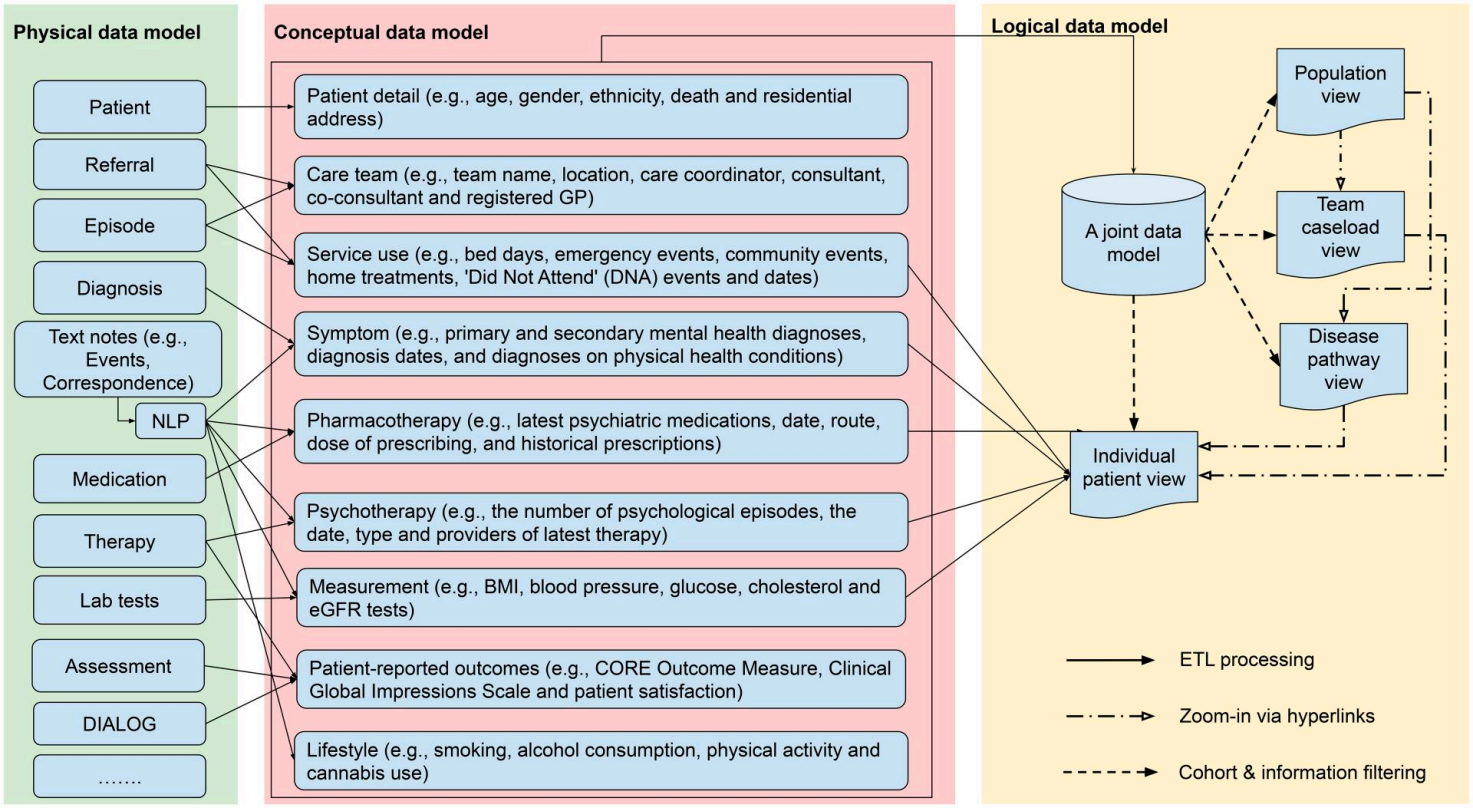
Diagram of VIEWER data models. The diagram visually depicts the transformation and organization of data across three models: (1) Physical Data Model: Represents the storage structure of source EHR data in physical data stores; (2) Conceptual Data Model: Illustrates the abstract structure of entity classes and their relationships, serving as the blueprint for data organization; and (3) Logical Data Model: Demonstrates semantic and logical relationships among entities, featuring hyperlinks and filtering mechanisms for efficient data access and analysis. The diagram also illustrates the progression of data through TEL processes, where the physical data model is converted into a common data model defined by the conceptual framework and then organized logically for streamlined use.

To meet the varying needs of different partners, we have crafted a comprehensive data model, encompassing a diverse array of patient attributes, clinical activities, and outcomes. As shown in Figure 2, we first identify relevant data sources within the source EHR, including both structured information and unstructured text data. These sources are then transformed and conceptualized into entity classes based on local settings and project-specific requirements.

The conceptual entities are then joined into a unified data table to consolidate the most recent information for each service user in the Trust. This consolidated data model undergoes daily updates and offers analytical and visual capabilities at various levels, ranging from a holistic population view to team-level caseload perspectives, disease-level pathway insights, and individual patient profiles. All these views are interconnected, allowing seamless navigation through embedded hyperlinks. For a granular patient-level examination, longitudinal data for time-dependent entities, such as service utilization, symptoms, treatments, and measurements, are incorporated, enabling the monitoring of patients’ health statuses and care plans over time.

### Interface design

The front-end interface of VIEWER consists of Web-based visualizations and dashboards developed using Kibana, an open-source data visualization tool available in CogStack.^31^ This contains 4 categories of dashboards, including “Population Health,” “Clinical Pathways,” “Caseload Management,” and “Patient Chart.” Given the stringent safety requirements, time-sensitive nature of clinical practice, and varying technical proficiency among end-users, we prioritize the following principles in the visualization design:

- Clarity: Ensure that visualizations remain clear, simple, and user-friendly, avoiding unnecessary complexity that could confuse end-users or increase training costs.
- Accuracy: Employ accurate graphs, labels, and representations to ensure precise representation of data in visualizations and facilitate cross-checks of information across different visualizations (eg, providing source text snippets for NLP extraction).
- Relevance: Only include relevant information that supports the intended message in the visualizations to avoid information overload.
- Integration: Ensure that the interface and content of visualizations are seamlessly aligned with clinical workflows and integrate seamlessly with EHR systems. Individual components are interconnected as a cohesive, self-contained system, for example, enabling flawless navigation from population-level visualizations to caseload and individual patient views.
- Interactivity: Add interactive features (eg, filtering and searching) when appropriate to allow users to explore the data further, gaining deeper insights based on their interests, beyond pre-defined information in default interfaces.
- Learnability and error recovery: To enhance learnability, employ consistent design elements such as icons, buttons, terminology, and structured layouts, while avoiding technical jargon. Additionally, incorporate default interfaces with “undo” options and accessible help guides to facilitate error recovery and ensure users can easily correct mistakes.

These dashboards are organized in a logical manner through a lightweight, configurable Web-based wrapper based on the Java Spring framework. When an authenticated user selects a dashboard via the navigation page (Figure S2), data are queried in Elasticsearch based on pre-defined filters in the dashboard, and the data are initially visualized in default formats. Users can interact with the interface by employing features like filtering, zooming, dragging, drilling down, selecting time ranges, and conducting full-text searches and queries to explore data and derive insights based on their interests. Smooth transition across dashboards for additional information and navigation from VIEWER to the source EHR system for actions are facilitated by hyperlinks based on Kibana scripted fields. Each visualization and dashboard is accompanied by short descriptions and banners that provide essential information about the data and visualizations.

Two versions of visualizations are available based on the use cases: the “clinical” version, which includes identifiable patient data for direct clinical tasks such as patient review and caseload management, and the “non-clinical” version, where patient identities are anonymized for non-clinical tasks like population based resource allocation, quality improvement, and demos/presentations. The de-identified data in the non-clinical version are generated using Kibana’s scripted fields and field anonymization features.

### Scalability

To ensure scalability of the system, we created a distributed computing infrastructure using Docker Swarm. Since all the data queried by end-users, search engine, and front-end interfaces are within CogStack, our focus is on enhancing the scalability of the CogStack instance. See Figure S1 for our distributed deployment of CogStack based on Docker Swarm on Azure. Processed data, extracted entities, audit logs, and metadata are stored in a distributed Elasticsearch cluster spanning 3 nodes, and hence new data are added and integrated seamlessly without system downtime. The distributed nature of this deployment also enables us efficiently manage and scale the system to accommodate a growing user base, increasing data volumes, and additional computational tasks over time.

### Data security and audit

This study was approved by multiple information governance bodies at SLaM, including the CRIS Oversight Committee for the development of the data model using de-identified data, the CogStack Oversight Committee for the VIEWER prototype utilizing identifiable data, where Secondary analysis of CRIS data was approved by Oxford Research Ethics Committee C (reference 18/SC/0372). Data Protection Impact Assessment and Clinical Risk Assessment have been conducted to ensure the development and deployment of VIEWER complies with the Trust’s data security and patient safety policies.

All data, infrastructure, and software used in VIEWER are securely housed within the Trust’s firewall and are accessible only to authorized users. Two user roles have been established to govern access to the front-end interfaces based on index-, document-, and field-level access control within the Kibana security plugins. A clinical role, providing access to identifiable patient data, is assigned to clinical staff who already have access to such data in the Trust’s EHR system. Conversely, a non-clinical role, providing access solely to the de-identified version, is assigned to non-clinical staff, such as managers and authorized researchers. Users with a clinical role can access both “clinical” and “non-clinical” versions of data, while users with a non-clinical role can only access “non-clinical” version. User activities, including logins and queries, are meticulously logged using Kibana audit plugins for audit purposes, ensuring that only relevant clinical information is accessible for a specific user.

## Results

### Patient characteristics

As of a recent census date on March 25, 2024, VIEWER ingested information for all patients (*n* = 409 870) who had a team episode or ward stay at SLaM, where 51 057 were active caseload, namely those currently managed by the Trust. Table 2 summarizes the patient characteristics for both active and inactive/discharged caseloads that have been integrated into VIEWER. Compared to the inactive caseload, patients in the active caseload tend to younger and have a higher proportion from the Black and Mixed ethnicity groups. Also, active caseload has a higher prevalence of severe mental health conditions, such as schizophrenia (ICD 10 code F2), bipolar disorder (F31) and major depressive disorder, and a higher proportion of living in the SLaM catchment area.

**Table 2.** Descriptive statistics of patient characteristics, grouped by active and inactive caseloads.

	All	Active	Inactive	*s*	*P*
Number of patients (%)	411 313 (100)	51 173 (12.44)	360 140 (87.56)		
Age	40.30 (18.55)	33.34 (19.11)	41.29 (18.25)	6.83E + 09	<.001
Gender				144.87	<.001
Female	212 244 (51.60)	26 869 (52.51)	185 375 (51.47)		
Male	198 068 (48.16)	24 065 (47.03)	174 003 (48.32)		
Unknown	1001 (0.24)	239 (0.47)	762 (0.21)		
Ethnicity				12 226.57	<.001
White	172 042 (41.83)	23 243 (45.42)	148 799 (41.32)		
Black	58 977 (14.34)	10 674 (20.86)	48 303 (13.41)		
Asian	20 129 (4.89)	2783 (5.44)	17 346 (4.82)		
Mixed	17 183 (4.18)	5062 (9.89)	12 121 (3.37)		
Other	23 368 (5.68)	3139 (6.13)	20 229 (5.62)		
Unknown	119 614 (29.08)	6272 (12.26)	113 342 (31.47)		
Diagnosis				9828.9	<.001
F0—Organic, including symptomatic, mental disorders	14 938 (3.63)	1256 (2.45)	13 682 (3.80)		
F1—Mental and behavioural disorders due to psychoactive substance use	31 621 (7.69)	3613 (7.06)	28 008 (7.78)		
F2—Schizophrenia, schizotypal and delusional disorders	19 415 (4.72)	5528 (10.80)	13 887 (3.86)		
F3—Mood [affective] disorders	42 123 (10.24)	3440 (6.72)	38 683 (10.74)		
F4—Neurotic, stress-related and somatoform disorders	44 523 (10.82)	4380 (8.56)	40 143 (11.15)		
F5—Behavioural syndromes associated with physiological disturbances and physical factors	13 376 (3.25)	1557 (3.04)	11 819 (3.28)		
F6—Disorders of adult personality and behavior	9189 (2.23)	1531 (2.99)	7658 (2.13)		
F7—Mental retardation	2675 (0.65)	412 (0.81)	2263 (0.63)		
F8—Disorders of psychological development	12 225 (2.97)	2226 (4.35)	9999 (2.78)		
F9—Behavioural and emotional disorders with onset usually occurring in childhood and adolescence	23 622 (5.74)	3768 (7.36)	19 854 (5.51)		
F99—Unspecified mental disorder	38 000 (9.24)	1827 (3.57)	36 173 (10.04)		
FX—No axis 1 disorder	19 969 (4.85)	2032 (3.97)	17 937 (4.98)		
Z—Factors influencing health status and contact with health services	44 533 (10.83)	4480 (8.75)	40 053 (11.12)		
Other illness	6391 (1.55)	246 (0.48)	6145 (1.71)		
Borough of residence				2780.83	<.001
Croydon	71 815 (17.46)	10 929 (21.36)	60 886 (16.91)		
Lambeth	66 144 (16.08)	10 498 (20.51)	55 646 (15.45)		
Lewisham	62 647 (15.23)	8625 (16.85)	54 022 (15.00)		
Southwark	62 434 (15.18)	7430 (14.52)	55 004 (15.27)		
Homeless	6521 (1.59)	438 (0.86)	6083 (1.69)		
Other	141 752 (34.46)	13 253 (25.90)	128 499 (35.68)		

Mann–Whitney *U* test is used to assess the statistical significance of differences between groups for numerical variables and the Chi-square test is used for categorical variables. Adjusted *P*-values with the Bonferroni correction were reported.

### Data characteristics

The VIEWER platforms efficiently ingest more than 1.7 million documents daily from source EHR systems, and support information retrieval and visualizations across more than 551 million documents for more than 1000 users across the Trust. Detailed characteristics of the data used in VIEWER are summarized in Table S1. Most time series data of patients were used to visualize patient charts, enabling clinicians to investigate longitudinal information for individual patients. In contrast, most visualizations for population, caseload, and pathway management are based on the information in the “Full caseload” table, as it reflects the most recent and comprehensive details of a patient’s care. The biggest table “Full caseload snapshots” contains time series of the “Full caseload” table, allowing the Trust or a team to monitor their service delivery over time.

### User interfaces

**Figure 3.**
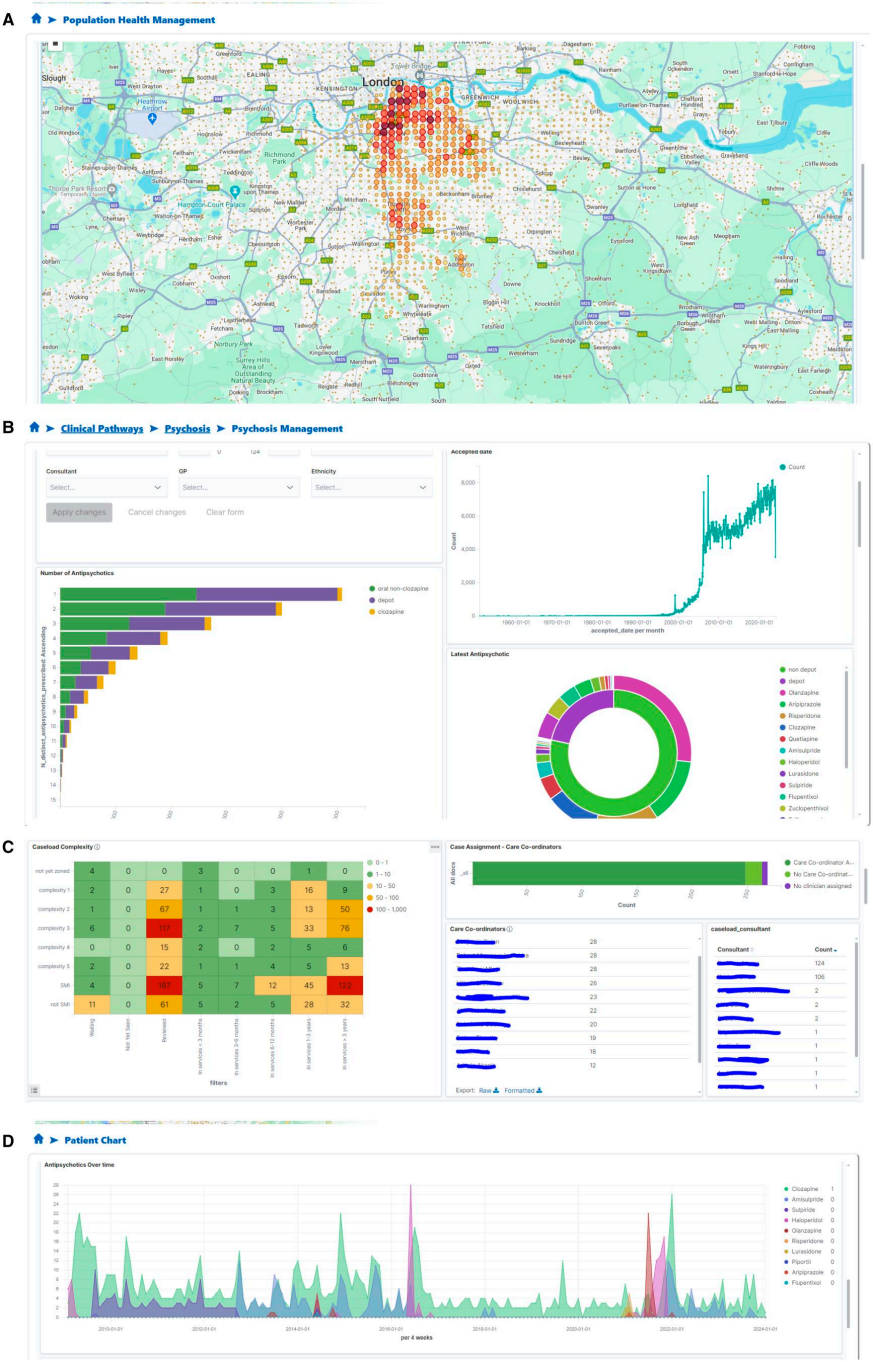
Screenshots for different dashboards in VIEWER. (A) Population Health View: A map displaying the residential locations of all active service users of the Trust, visualized as geographic markers to illustrate population distribution; (B) Clinical Pathways View: A graphical representation of anti-psychotic medication use patterns among patients diagnosed with psychosis, highlighting medication trends and usage; (C) Caseload Management View: A heatmap chart stratifying case complexity within a care team’s caseload, categorized by duration of service use, care coordinators, and consultants to provide workload insights; and (D) Patient Chart View: A time-series graph showing anti-psychotic prescriptions over time for an individual patient, enabling detailed review of medication history.

As shown in Figure 3, VIEWER provides Web-based interactive, problem-oriented visualizations of patient data through multiple perspectives, including:

- Population health: This category of visualizations aim to offer a comprehensive overview of the whole population served by the Trust, encompassing demographics, geographical distribution, diagnoses, outcomes, and care teams. It provides multidimensional filters that allow users to delineate sub-populations and discern specific needs, such as identifying regions with elevated incidents of new cases, those with stretched resources, and inequalities in service access.
- Clinical pathways: This category includes a set of dashboards designed to facilitate proactive care through a multidisciplinary approach to the assessment, diagnosis, and treatment of individuals experiencing symptoms associated with specific mental disorders, such as psychosis and bipolar disorders. Visualization components within this category include identifying cohorts of patients who have not received interventions and may benefit from them based on historical treatments and responses. It also allows to identify patients who need a medication review based on their side effect profiles and those showing early signs of relapse or non-adherence to treatments, and assess who has met national care standards, including prescribing, psychological therapy, and physical health. For discharged patients, it facilitates coordination with their primary care physician to anticipate and prevent relapse, as well as reduce reliance on crisis pathways. Additionally, it provides an overview of the entire cohort with a specific diagnosis, including both those currently under care and those who have been discharged.
- Caseload management: This category focuses on enhancing caseload management within a clinical team. It involves visualizing uneven distributions of case risk and complexity across team members, identifying factors contributing to risk/complexity, highlighting potential indicators of unmet needs (such as crisis care, patients’ clinical outcomes, and their satisfaction with services), monitoring care continuity during leaves or absences. This also enables the MDT within the team and the broader system to identify where limited resources should be allocated to achieve better outcomes.
- Patient chart: This category encompasses visualizations that provide detailed, individual-level information, offering insights into the current state of a specific patient (eg, recent interventions and outcomes) as well as historical data on interventions and outcomes over time. By integrating longitudinal data from multiple entities, clinicians can efficiently comprehend the overall care trajectory of a given patient. This facilitates cross-checks of care data against clinicians’ own understanding and enables the identification of personalized needs and care plans for individual patients.

### Clinical applications

#### Physical health monitoring

The first use case of VIEWER was to support annual physical health check for all active patients within the Trust, particularly for those living with severe mental health conditions or undergoing long-term psychiatric medication treatment. Compared to the general population, people with mental health conditions often face poorer physical health,^41^ which has contributed to reduced quality of life and a shortened life expectancy by 10-20 years for people with severe mental health conditions.^42^^,^^43^ This disparity is partly due to the potential side effects of psychiatric medications, such as weight gain, metabolic syndrome, cardiovascular risks, and other chronic conditions.^44^^,^^45^ To enhance overall health and decrease premature mortality among this group, clinical guidelines recommend annual physical health checks to promptly identify and manage physical health conditions (https://www.england.nhs.uk/long-read/improving-the-physical-health-of-people-living-with-severe-mental-illness/). These assessments typically include the evaluation of multiple parameters, including blood pressure, body mass index (BMI), blood glucose or HbA1c levels, lipid profile, smoking status, alcohol consumption, and lifestyle factors like diet and physical activity levels, which can be time-consuming.

**Figure 4.**
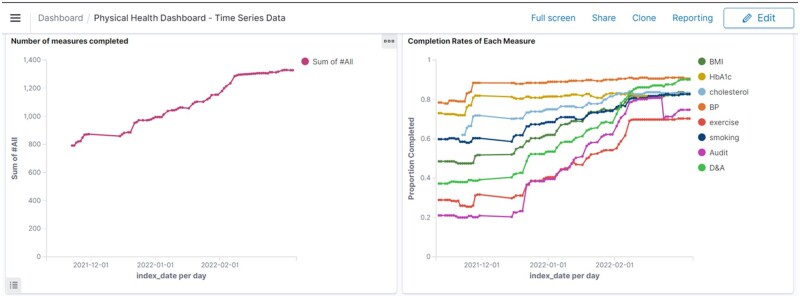
A graph showing the number of measures completed and the completion rate for each measure in the annual physical health check, tracked over time for patients managed within a specific care team.

VIEWER has served as the primary system supporting a team of 6 members in overseeing physical health checks for the entire active caseload of over 51 000 patients within the Trust. VIEWER provided a comprehensive overview of physical health checks by leveraging NLP-based information extraction methods. Through interactive visualizations, team members can readily identify patients who require a physical health assessment. Each identified patient is provided with a hyperlink that directs clinicians to their profile within the EHR system, facilitating actions such as scheduling a check or contacting GPs for additional information. After completion, these checks are recorded in the source EHR system and then incorporated into VIEWER. This systematic approach has streamlined the check process and resulted in an increase in completed assessments. For example, Figure 4 shows the rise in the number of measures completed by the team after adopting VIEWER as a systematic approach within a short period of 2 months.

#### Medication review

VIEWER’s second use case was to facilitate medication review for 164 819 patients with documented prescriptions for psychiatric medications, including antipsychotics, antidepressants, and mood stabilizers. Many individuals in secondary mental health services take psychiatric medications, often for long periods.^46^ Regular reviews are essential for optimizing treatment outcomes, improving adherence to prescribed medications, and minimizing adverse effects.^47^ This process requires a thorough assessment of all medication records within the entire EHR. Since most prescribing information and related contextual details (eg, adverse reactions and non-adherence) are recorded in unstructured text such as clinical notes and correspondences between healthcare professionals (eg, mental healthcare specialists, pharmacists, and GPs), each review typically takes hours to complete in the local Trust.

VIEWER enables to reduce the review time through user-friendly visualizations of a comprehensive medication data model, which integrates medication information extracted from both structured data and unstructured text using NLP techniques. In a preliminary evaluation of this new method, a pharmacist reviewed 3 medication cases previously completed by another pharmacist. For each case, a comparison was made between the medications identified by the human pharmacist and those generated by VIEWER. The results showed that 100% (*n* = 20) of the antipsychotics, antidepressants, and mood stabilizers identified in the human reviews (*n* = 3) were also present in the VIEWER-generated lists. VIEWER also identified 4 extra medications not mentioned in the human reviews. Of these, 75% (*n* = 3) were treatments that had been considered but not started, and 25% (*n* = 1) was an antidepressant that had been trialed. Furthermore, the time spent per review using VIEWER was reduced from 1-2 hr to 10-20 min.

#### Caseload management

The last use case of VIEWER discussed here focuses on enhancing caseload management to meet the requirements set by the Care Quality Commission (CQC), an independent regulator of health and adult social care in England. Caseload management involves various aspects of a patient’s mental health needs, ranging from medication management, psychotherapy, social support, and resource allocation. Effective caseload management enables healthcare providers to improve efficiency in resource use, reduce waste, and maximize patient outcomes. Table 3 lists exemplar tasks supported by VIEWER in the local mental health Trust. Since the Trust’s EHR system was primarily developed for recording clinical data at the point of care for individual patients, it has limited capability for retrieving information for a group of patients. These tasks thus often require intensive manual work to review each patient’s records and aggregate the data. This process is not only time-consuming but also introduces inconsistencies in data collection, analysis, and recording.

**Table 3.** Caseload management tasks supported by VIEWER.

Task	Purpose	Examples
Prioritization and plan	Evaluating the needs of each patient and prioritizing care based on the severity and urgency of their conditions	− Identify Red-Zone patients.
		− Locate accident and emergency (A&E) attendances.
		− Assess complexity and risk factors for a patient.
		− Plan outreach care such as home visits based on geography.
Resource allocation	Effectively allocating resources, including staffing and services, to ensure that all patients receive the appropriate level of care.	− Review caseload for each care coordinator.
		− Allocate new referrals and assign caseload across care coordinators based on complexity, geography/GP catchment area, rather than number.
		− Detect unassigned patients.
		− Identify patients eligible for earlier discharge based on complexity, risk and use of crisis services.
Coordination of care	Ensuring that patients receive comprehensive care by coordinating services among various healthcare providers, such as psychiatrists, therapists, primary care physicians, and social workers.	− Facilitate multi-disciplinary team (MDT) in-reach opportunities to support care coordination by psychology, physical health, occupational therapy social care etc., based on DIALOG outcomes and individual’s goals and care planning actions.
		− Support of GP link-working as part of community transformation by identifying service users under a particular GP in joint meetings with Primary Care Community Mental Health Teams (PCMHTs) or direct liaisons.
		− Identify social determinants of health outcomes, such as housing stability and employment status, and involve other services and agencies.
Monitoring and follow-up	Regularly reviewing patient progress and adjusting treatment plans as needed to ensure the best outcomes.	− Track previous diagnoses and medical history.
		− Monitor changes of complexity and risk over time.
		− Medication administration follow-up.
Documentation and reporting	Maintaining accurate records of patient interactions, treatment plans, and outcomes to track progress and inform future care decisions.	− Team caseload summary.
		− Review of patient outcomes.
		− Improve the documentation of key performance indicators (KPIs).

By integrating data aggregation processes in data ingestion pipelines and filters within Kibana visualizations, VIEWER allows team members to access consistent and unified information about their caseloads. Also, interactive data visualizations in VIEWER enable users to complete these tasks with just a few clicks, significantly improving efficiency. For example, team leaders need to regularly review the number of patients under each care coordinator during supervision, a task that typically takes at least 20 min per review. In contrast, this can be accomplished with a single click of a filter for a team name or a care coordinator’s name in VIEWER. Additionally, VIEWER’s rapid development capability has enabled the Trust to meet CQC requirements and deliver results for an annual inspection within just 6 months.

### Usability and acceptability

**Figure 5.**
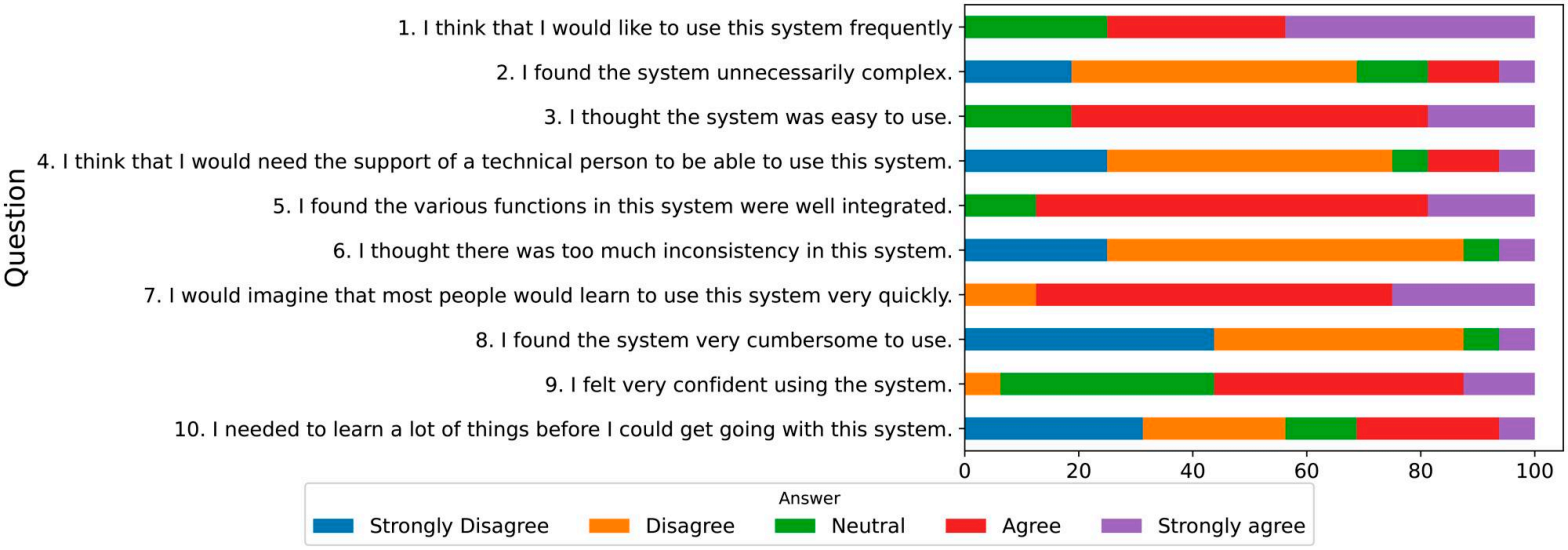
A chart displaying the proportions of different responses to each question in the SUS (System Usability Scale) survey.

To assess the usability of VIEWER, we used the standard System Usability Scale (SUS), which consists of a 10-item questionnaire with 5 response options ranging from “Strongly agree” to “Strongly disagree”^48^ during its early development stage. Sixteen users completed the questionnaire, and the overall score was 74/100, indicating “good” usability. Figure 5 presents the detailed responses for each SUS question from the 16 respondents. The positive scores are further supported by the respondents’ feedback in Table S2. Among the responses, 4 users scored the system over 80, indicating potential promoters of the system, while 2 users scored below 55, making them potential detractors. Both potential detractors provided free-text responses stating that they found the system useful and had used it to improve patient care but expressed a need for more training.

**Figure 6.**
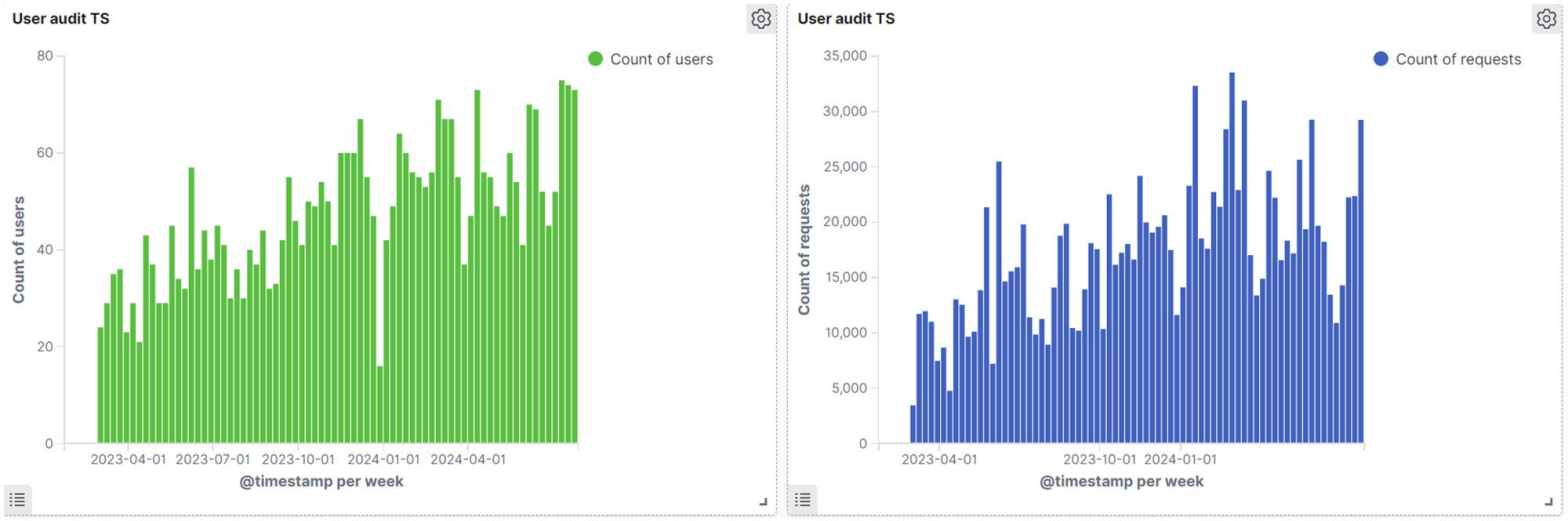
A graph showing the number of users and requests per week over the past two years.

The acceptability of VIEWER is illustrated by its wide adoption within the Trust. Over 1000 clinical and managerial staff members, out of approximately 5000 total staff (including both clinical and non-clinical roles), have used the system over the last 2 years. VIEWER has handled more than 147 million data requests, with user activity likely to increase, as shown by the trends in Figure 6. Due to these positive outcomes, the Trust and the Maudsley Charity have recently committed a 4-year funding to operate the system as a business-as-usual service, aiming to achieve broader and longer-lasting impacts.

## Discussion

This paper presents the design, development, implementation, and evaluation methods of VIEWER: a lightweight, extensible toolkit for rapid creation and deployment of clinical information extraction, analysis, and visualization to enhance data accessibility in various aspects of healthcare delivery. VIEWER leverages advanced NLP-based information extraction methods to derive clinical insights from diverse EHR data and employs interactive visualizations to effectively communicate these insights, enabling informed decision-making at the point of care. Designed to complement, not replace, existing EHR components, VIEWER is built primarily on extensive pre-existing but researcher-focused capability (ie, CRIS) and open-source technologies such as CogStack, offering a cost-effective enhancement to the functionalities and intelligence of legacy EHR systems. This system has demonstrated valuable impacts in various real-world clinical applications within a large mental health Trust.

While many clinical information retrieval and visualization tools have been developed previously, most have either focused on processing structured tabular data^8^ or specific tasks (eg, individual patient information summarization^7^ or cohort search^16^) and data related to certain diseases.^18^ VIEWER advances this field by employing scalable, distributed, NLP-based information extraction pipelines that enable comprehensive search and analysis of both structured and unstructured information for all service users within a Trust. While our implementation utilizes in-house NLP applications for information extraction, VIEWER supports the integration of open-source or custom NLP models as plugins (https://github.com/CogStack/CogStack-Pipeline). Moreover, by leveraging interdisciplinary expertise and participatory design approaches, VIEWER provides a user-friendly interface that seamlessly integrates with existing EHR systems and enables information presented in a useful manner. This capability effectively addresses a broad spectrum of real-world applications, ranging from population health management and service/specialist-specific caseload optimization to personalized care plans for individual patients. The interactive visualizations also allow for customization to suit local clinical specialties, workflows, and preferences.

VIEWER also offers high potential for interoperability, allowing for directly deploying on top of existing EHR systems or incorporating into clinical research platforms to facilitate the translation of research into practice. In our implementation, we leveraged the clinical research infrastructure in our institution and use VIEWER to facilitate the transformation of research outcomes, particularly in data linkage and NLP pipelines within CRIS, into real-world clinical practices. As a result, the extensive set of clinical information extraction and NLP pipelines, developed and refined through multiple CRIS research projects over the last decade, has now been applied to inform clinical decisions directly at the point of care. This also enhances the accessibility of clinical research datasets and enables large-scale studies at the population level, for example, examining outcome disparities across populations.^35^ However, when a CRIS-like system is absent, CogStack, the backbone component used for data ingestion and visualization in VIEWER, provides a set of open-source tools capable of parsing text content from binary documents (eg, PDFs, scanned images, and Word files) and NLP packages for extracting medical information from clinical text (https://github.com/CogStack/CogStack-Pipeline). Indeed, CogStack has been deployed at multiple NHS sites, including both mental and physical healthcare providers in the United Kingdom and beyond.^31^ This offers a high degree of flexibility for easy customization when integrating VIEWER with various EHR systems directly.

Another notable strength of VIEWER is its extensibility and customizability. Although originally developed within mental healthcare settings, the VIEWER framework is vendor-agnostic and capable of importing data from various relational databases and external systems such as sensor devices and fitness monitors. While different settings have varying priorities and workflows, our data model design closely aligns with the Observational Medical Outcomes Partnership Common Data Model, an open community data standard for observational health data sciences and informatics,^49^ with modifications tailored to local settings and project-specific requirements. Also, our interface design adhere to well-established health informatics design methods^34^^,^^49^ and are general to cover key components in clinical practice. This versatility enables VIEWER to be effectively applied in other settings to enhance data usage and supporting informed clinical decision-making. Also, VIEWER supports extensive customization options, including visualization coding tools like Vega^50^ and graphical user interfaces, as well as integration with external libraries for predictive and statistical learning algorithms available in languages such as Python and R. This customizability empowers both technical and non-technical users to design and refine data visualizations and analytical dashboards. In fact, most visualizations in VIEWER were developed by clinicians rather than technicians, which has enhanced its clinical relevance and facilitated its rapid adoption in practice.

The insights gained and factors contributing to VIEWER’s success have led us to make the following recommendations for designing CDSS.

1. Interdisciplinary collaboration: As highlighted previous guidelines,^51^ the success of CDSS hinges on their ability to tackle real-world challenges faced by clinicians. To ensure this, it is essential to co-design a system with input from a broad range of partners with interdisciplinary backgrounds, particularly clinicians and governance committees. From our experience in implementing VIEWER, this collaborative approach not only enhances the understanding of practical challenges and helps identify relevant solutions more effectively, but also fosters the involvement of key partners as part of an iterative process to support the creation of practical models that are more likely to be adopted effectively.
2. Complexity and usability: As identified in previous studies, complexity is a major barrier to the adoption of CDSS, which often causes a temporary decline in productivity as users navigate the learning curve of a new system.^52^ This aligns with our observations when implementating VIEWER. To ensure high adoption rates and maximize the system’s overall impact, CDSS should strike a good balance between simplicity and complexity, so that it can effectively model real-world scenarios but remain user-friendly and easily interpretable for its intended users.^51^ We also find that when achieving simplicity is infeasible, providing adequate training and support is essential to manage the inherent complexity. Moreover, interface components should be organized to fit users’ workflows^53^ and maintain consistent styles to minimize the learning curve.
3. Multi-dimension integration: Integrating with local EHR systems and aligning with users’ workflows are critical for enhancing a CDSS’s accessibility and usability. In our implementation of VIEWER, users’ most common question was difficulty in logging into the new system. This problem was largely resolved by integrating Single Sign-On^54^ authentication with the local EHR. However, deep integration of a CDSS with existing EHR systems can be challenging due to the required technical infrastructure and staff expertise.^55^ Therefore, it is important to integrate all components of a CDSS into a cohesive, self-contained system within a sandboxed environment to ensure a smooth user experience during the early stages of implementation. Also, due to security and safety concerns, real-time interactions (eg, information entry and modification) are often restricted from being pushed back to the EHR from the CDSS. Embedding a hyperlink to allow users to seamlessly navigate between the CDSS and the EHR system can effectively address this issue.
4. Iterative feedback: Regular performance monitoring and evaluation are important to ensure that a CDSS meets clinical needs. In the VIEWER system, we developed several visualizations and dashboards to monitor the treads of clinical outcomes over time. These monitoring panels not only enable consistent measurements of performance but also feature a user-friendly, interactive interface that allows customization of parameters to effectively address local needs for performance analyses. This creates an electronic audit and feedback cycle, which starts from informing goal setting, streamlining interventions to automatically collecting and analyzing data, leading to iterative feedback to optimize performance. These feedback cycles are also useful for motivating behavior changes and system adoption, particularly when the feedback directly supports clinical behaviors.^56^
5. Transparent communication: A new system may initially fall short of users’ expectations and require their engagement for further improvement. While promptly responding to users’ requests and feedback is vital to maintain their engagement, active listening and transparent communication are essential to maximize the outcomes of such engagement. In our interactions with VIEWER users, we highlighted not only the strengths of our solutions but also their limitations, encouraging users to contribute to resolving these issues and refining the system. This approach fosters a sense of “ownership” over the feedback process, making users feel more invested in the improvements. Such ownership-driven feedback leverages users’ autonomy and internal motivation to enhance the system, rather than feeling subjected to external policies or directives.^56^

This includes incorporating robust privacy-preserving techniques, such as differential privacy, and ensuring that all stakeholders (clinicians, patients, and developers) are properly informed about how their data are being used and processed. Ethical considerations, especially regarding mental health data, will be crucial as we continue to develop and scale such technologies.

VIEWER and this study of its use also have limitations and require further improvement. First, although a range of NLP applications have been used to extract useful information from clinical text, which contains the majority of patient information, this task remains challenging. The primary difficulties stem from the extensive use of medical terminology, grammatical inaccuracies, and the sensitivity of clinical data, which results in limited availability of data for training large language models.^57^ Our users have reported that temporality (distinguishing past, present, or future events), certainty (differentiating confirmed and hypothetical events), and negation are the most common sources of errors in NLP extraction results, highlighting areas that require further improvement.^58^ As a contingency solution, VIEWER provides text snippets that offer complete context for each extracted event, allowing users to interpret and validate the NLP results effectively. Moreover, real-time extraction of information and aggregation of NLP-extracted information to construct care trajectories proves useful but also require further development. Given the sensitive nature of mental healthcare data, it is essential to prioritize patient confidentiality and privacy in the development of these technologies. This requires strict adherence to ethical guidelines, such as data anonymization, and compliance with regulations like the GDPR and the NHS Data Security and Protection Toolkit.

Second, beyond functionality, the sustainability of healthcare informatics solutions—encompassing aspects such as maintainability, scalability, and interoperability—is crucial for enhancing the quality and affordability of healthcare delivery. To fully understand the benefits and limitations of VIEWER, it is important to compare it with other open-source or commercial solutions that offer similar capabilities. A more extensive comparison, based on longer and broader usage, would provide valuable insights into whether the findings are widely applicable. Third, VIEWER has primarily been used by clinicians at SLaM. However, it would be beneficial to make VIEWER’s patient chart features available to individual patients, enabling them to monitor their own health metrics and input relevant health data. To explore this, we would need authorization to grant patient access to the system, as well as technical support and data integration services to connect with other patient portals.

Finally, VIEWER has only been deployed at SLaM so far, and thus our evaluation focused on findings in a single institution. However, as VIEWER was designed to be EHR-agnostic, porting it to another EHR system is feasible. We are eager to deploy VIEWER in other institutions and assess the generalizability of our findings. Moreover, this paper has primarily focused on outlining the technical components and development process of VIEWER, and our current evaluation relies on observational research methods. To formally assess the effectiveness of our informatics intervention and determine the factors contributing to its success, such as the types of use cases and participant characteristics, including backgrounds, demographics, and areas of expertise, across various teams and settings, randomized controlled trials are needed.

## Conclusion

As EHR data rapidly increases, there is an urgent need to enhance data accessibility and interpretability across various aspects of healthcare delivery, such as population health management, caseload optimization, patient monitoring and personalization of care. Through interdisciplinary collaboration, we developed VIEWER: a cost-effective, open-source, and extensible toolkit designed for the rapid design, development, and implementation of clinical information retrieval, analysis, and visualization to meet this need. Deploying VIEWER in one of the largest mental healthcare provider in the United Kingdom further enables us to explore the use of patient data visual-analytics as an informatics intervention to enhance patient care in real-world clinical settings. Our implementation offers valuable insights into collaborating with clinicians to iteratively refine and optimize the interface and content of a health informatics solution for better patient care.

## Supplementary Material

ocaf010_Supplementary_Data

## Data Availability

All data utilized in this study are derived from patient records within SLaM and are not publicly accessible. Details about the software and tools employed in the research are provided within the article, including access links where available or upon request.
