# Supplementary material for: VIEWER: an extensible visual analytics framework for enhancing mental healthcare
Source: J Am Med Inform Assoc. 2025 Jan 23;33(1):144–58. doi: 10.1093/jamia/ocaf010 (PMC12758470; doi:10.1093/jamia/ocaf010)
Supplement: ocaf010_Supplementary_Data [file ocaf010_supplementary_data.pdf]

Supplemental Information

VIEWER: an extensible visual analytics framework for enhancing mental healthcare

Contents

List of Figures

S1 Docker-based deployment of CogStack 2
S2 VIEWER home page 3

List of Tables

S1 Descriptive statistics of data models 2
S2 User feedback 3

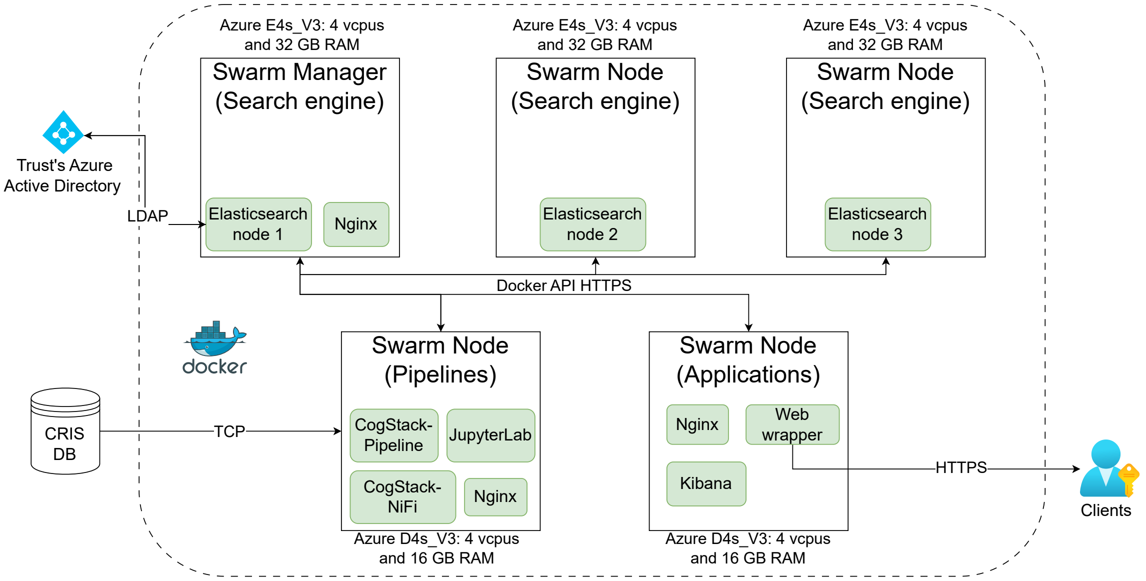

Figure S1: A distributed deployment of CogStack based on Docker Swarm. Five Azure virtual machines host three types of containerised micro-services: 1) data storage, index and search engine, 2) data ingestion and analytical pipelines, and 3) interface applications.

Table S1: Descriptive statistics of data models.

Table/Index	Description	Column Count	Row Count
BMI catalogue	BMI values of each patient over time, including those recorded in both structured fields and text notes extracted using NLP methods.	11	399,277
Psychology episodes	Psychological therapy episodes for each patient over time.	39	84,224
CORE_OM scores	CORE Outcome Measure (CORE-OM) scores for each patient over time.	70	103,528
Medication catalogue	Each patient's medication records over time, including structured fields and records in text notes extracted using NLP.	29	18,929,237
Test results	Lab results of patients over time.	14	704,327
Contact catalogue	A patient's service contacts (e.g. bed days, community care and emergency services) over time.	17	17,373,156
Clinician caseload	All patients that a staff member is currently caring for.	20	46,892
Diagnosis catalogue	Diagnosis records of each patient over time.	18	1,061,482
Full caseload	Caseload episodes for each patient, including latest information about patient details, referrals, care teams, diagnoses, treatments, and outcome observations such as lab tests and DIALOG.	625	1,611,007
Full caseload snapshots	All snapshots of the caseload table for monitoring data over time.	625	510,992,515

Table S2: Respondent feedback on the overall use of VIEWER.

"It has helped with improving patient's physical health care as it is able to identify what physical health checks a client has not had therefore we are able to identify these easier and put in place interventions quicker."
"It has definitely stimulated interest to use in practice. It is a helpful tool in presenting, reviewing and auditing."
"It definitely helpful in community transformation project, in identifying the most vulnerable patients and demographic. The GP practice which to focus attention as we move into neighbourhood workings, the number of patients on depot medication where to support in the transition to primary care."
"Helps to identify physical health needs and help determine what interventions need to be offered and signposted to external agencies. Helps identify what anti-psychotics have been offered and whether clozapine should be considered as an option. It helps collating information on ePJS (the Trust's EHR system) which saves time - CC's don't have to manually scrutinise notes."
"I was using this to visualise ethnicity data to support a policy lead in conducting an Equality Impact Assessment on the Clozapine policy. This will hopefully inform further ethnicity analysis, discussion and action in relation to this policy and its implementation."

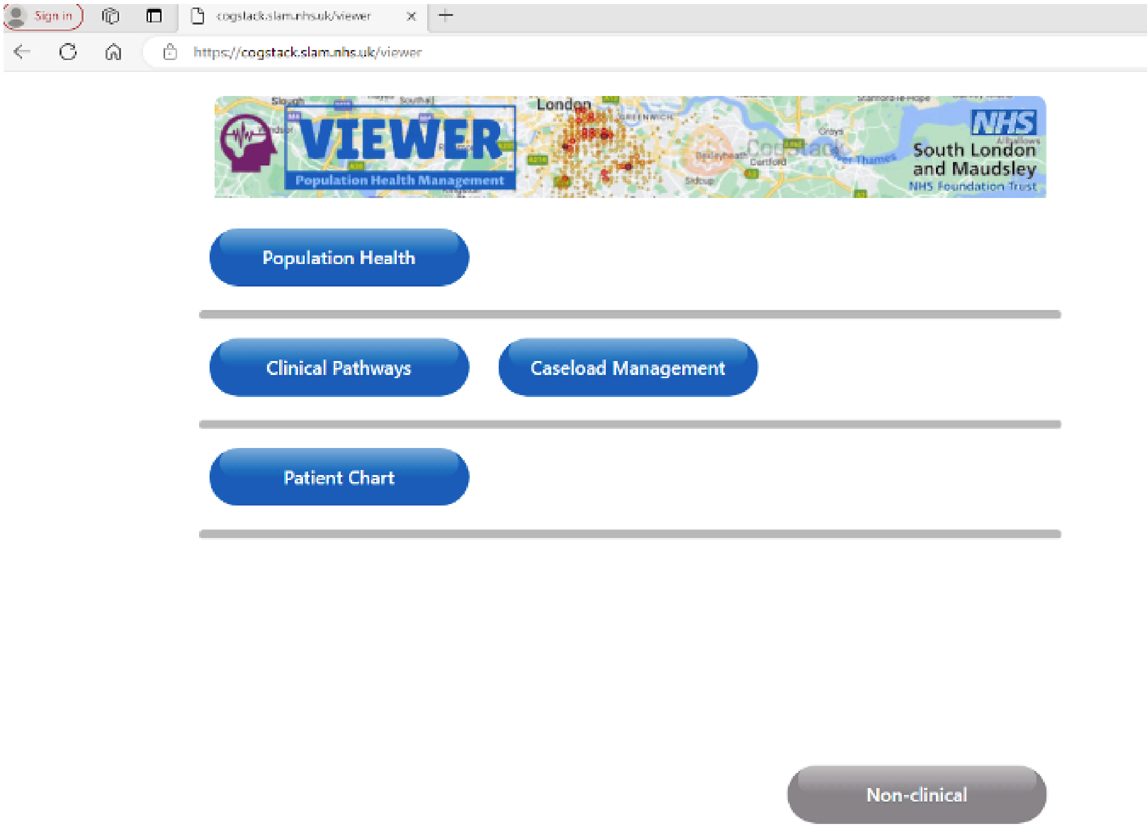

Figure S2: VIEWER home page. Dashboards are organised into four main categories, namely "Population Health", "Clinical Pathways", "Caseload Management" and "Patient Chart". Each category employs a hierarchical structure to facilitate clear navigation among sub-sections, such as different diagnoses under "Clinical Pathways". The "Non Clinical"/"Clinical" button in the right bottom allows users to switch between de-identified and identifiable versions.
